# Searching for a needle in a haystack—ambulatory research in Parkinson’s disease in Germany

**DOI:** 10.3389/fnagi.2026.1820262

**Published:** 2026-06-10

**Authors:** Ingmar Wellach, Dirk Becker, Kristina Schmidt, Isabel Doblinger, Susann Eichler, Lisa Hillmer, Honorine Atanga, Christoph Redecker

**Affiliations:** 1 Association for Quality Development in Neurology and Psychiatry (QUANUP e.V.), Hamburg, Germany; 2 Department of Neurology and Neurogeriatrics, Medical School and University Medical Center OWL, Hospital Lippe, Bielefeld University, Lemgo, Germany; 3 Neurology and Psychiatry Practice Hamburg Walddoerfer, Hamburg, Germany; 4 Evangelical Amalie Sieveking Hospital, Hamburg, Germany; 5 Department of Science and Academic Medicine, Medical School and University Medical Center OWL, Hospital Lippe, Bielefeld University, Detmold, Germany; 6 Neuropraxis Detmold, Detmold, Germany

**Keywords:** ambulatory research, ambulatory standard care measures, Germany, Parkinson’s disease, survey

## Abstract

**Background:**

Parkinson’s disease (PD) is a prevalent neurodegenerative disorder with a rapidly increasing incidence worldwide and complex treatment requirements. It is predominantly treated on an outpatient basis. However, detailed clinical data from outpatient practice remain scarce. We therefore set out to evaluate PD care practices in the outpatient setting. As preliminary work for a pilot project, this report represents the first systematic exploratory survey to assess outpatient treatment patterns in Germany. The aim of this survey is to document the current outpatient treatment approaches for PD, to identify potential gaps in the existing clinical guidelines, and to highlight the lack of outpatient-specific evidence.

**Methods:**

A cross-sectional baseline survey was conducted online using a standardized questionnaire among neurologically specialized outpatient practices in Germany. Serving as a baseline assessment of the regional status quo of current care practices, the survey forms the foundation for the subsequent multicenter pilot study AmParkReg, into which these results will later be integrated.

**Results:**

The survey revealed a high degree of homogeneity in routine clinical assessments across various practice types with an emphasis on psychomotor examination, particularly rigidity assessment, reflecting established dopaminergic treatment monitoring. However, the integration of non-motor symptom assessment and comprehensive multimodal diagnostics remains limited. Time constraints and regional variations in practice warrant further investigation. Methodological limitations include regional sampling bias and the lack of temporal data.

**Conclusion:**

These findings underscore the urgent need for ambulatory-specific, evidence-based care standards in PD. The development of longitudinal, multisectoral studies and the strengthening of ambulatory care guidelines are critical for optimizing patient outcomes. This work provides foundational data to refine ambulatory management and fosters the integration of precision approaches into routine PD care in Germany.

## 1 Introduction

Parkinson’s disease (PD) is a progressive neurodegenerative disorder characterized by an α-synuclein-associated loss of predominantly dopaminergic neurons throughout the brain. In addition to the typical motor symptoms, which are attributable to degeneration of nigrostriatal afferents from the substantia nigra pars compacta, affected individuals also suffer from a variety of non-motor symptoms involving other neurotransmitter systems in different brain regions, e.g., autonomic disorders, depression, or dementia (Venda et al., 2010; Yusuf et al., 2025).

It is estimated that more than 400,000 people in Germany are currently affected, and due to population aging, a significant increase in the prevalence of PD is expected in the coming years (Heinzel et al., 2018). Some international studies even suggest a pandemic-like increase in the number of affected individuals worldwide (Dorsey et al., 2018; Dorsey and Bloem, 2018). PD is characterized by a high symptom burden and a complex, life-changing disease course (Müller et al., 2013; Jankovic and Tan, 2020; Port et al., 2021; Tarolli et al., 2020). At the same time, the disease causes considerable direct and indirect costs, placing a heavy burden on the healthcare system (Chaudhuri et al., 2024). Its complexity poses major challenges for both the patients’ families and healthcare providers, as care must be delivered in both outpatient and inpatient settings over decades (Soares et al., 2023; Tönges et al., 2019; Donzuso et al., 2025; Oguh and Videnovic, 2012).

The German healthcare system is a mixed system consisting largely of statutory health insurance (public) with a smaller proportion of privately insured patients. It is divided into inpatient and outpatient care, with a clear historical division of responsibilities (IQWiG, 2006). The inpatient sector primarily covers acute and complex interventions, whereas the outpatient sector provides continuous medical care and therapy throughout the entire course of the disease (Buhmann et al., 2016; Kleinholdermann et al., 2025; Temlett and Thompson, 2006; Weise et al., 2024; Prell et al., 2020). Outpatient neurology care is predominantly delivered in independently operating practices within the statutory health insurance framework.

Although treatment recommendations and “best practice” guidelines for PD have been available in Germany for many years, their implementation remains inconsistent and incomplete (Binder et al., 2018; Timpel et al., 2022). The reasons for this have not yet been conclusively investigated and can therefore only be hypothesized. One possible reason is that current recommendations do not distinguish between different treatment settings. Further obstacles may arise from characteristics of the German healthcare system, including structural differences between inpatient and outpatient sectors, limited reimbursement mechanisms, time constraints in outpatient neurology, and regional disparities in care availability.

As a consequence, depending on regional care structures, patients are treated by a range of providers, including general neurologists, movement disorder specialists, outpatient clinics, and general practitioners, often without corresponding guidelines for diagnosis and therapy (Temlett and Thompson, 2006; Weise et al., 2024; Prell et al., 2020).

Guidelines and clearly structured framework conditions for inpatient interventions in PD have been in place for several years, particularly for complex PD treatments, such as the German Parkinson-Komplex-Behandlung (PKB) or Parkinson-Komplex-Therapie (PKT) (Richter et al., 2019; Scherbaum and Tönges, 2024). In accordance with reimbursement regulations under the German diagnosis-related group (DRG) system, the DRG category B49Z for complex inpatient PD treatment requires documentation of regularly administered therapies, in particular speech therapy, occupational therapy, and physiotherapy (Ebersbach, 2008). However, these inpatient therapies (e.g., according to DRG B49Z) are highly resource-intensive multimodal treatment programs and entail a risk of frequent hospital admissions, thereby posing major challenges at the interfaces between outpatient and inpatient care sectors (Oguh and Videnovic, 2012; Buhmann et al., 2016).

Day clinic treatment options are evidence-based and effective but cannot fully meet the increasing demand for care and do not substitute for continuous outpatient care (Fründt et al., 2018; Buhmann et al., 2026). In rural areas, complex disease progression and gaps in care often lead to avoidable hospital admissions, underscoring the importance of a well-developed outpatient infrastructure (Timpel et al., 2022).

Although most scientific data on treatment and disease progression in PD derive from the inpatient sector, long-term data from outpatient care are largely lacking. This represents a considerably under-researched field to date, with far-reaching medical, social and health policy implications (Gros et al., 2025). Scientific research on PD is therefore predominantly based on data from inpatient settings, particularly university hospitals (Gros et al., 2025).

Despite the central role of outpatient care in providing long-term support for people with chronic conditions, systematic scientific analyses in this area remain scarce. This is largely attributable to limited human, infrastructural, and financial resources in private practices, which are generally not equipped to conduct long-term studies. Methodological challenges in outpatient research include data management issues, heterogeneous documentation standards, and the lack of longitudinal study designs. Moreover, routine data rarely capture detailed clinical information, such as symptom severity, non-motor complications, or treatment adherence.

Consequently, outpatient care remains an under-investigated domain, even though most patients with PD are treated in this setting over extended periods of time. The provision of continuous, guideline-adherent care is often challenging, particularly in regions where access to specialized neurological care is limited and patients are managed primarily by general practitioners. This situation poses specific risks, especially when prescribed medication is not continued consistently after hospitalization, leading to gaps in care (Himmel et al., 1996). The present approach is intended as a first step toward identifying scientific, methodological, and systemic challenges in outpatient PD care and, based on these findings, formulating further research questions arising from routine outpatient treatment.

This survey was designed and coordinated by the Association for Quality Development in Neurology and Psychiatry (QUANUP e. V.) and conducted by the Hamburg Walddoerfer Practice for Neurology and Psychiatry, a certified PD specialist practice. The structured survey of 21 practices in the Hamburg metropolitan region and beyond is described below, and its results are subsequently presented and discussed.

## 2 Materials and methods

### 2.1 Survey design

The survey described above uses a cross-sectional design with the aim of capturing, for the first time, previously undefined standards of care in outpatient PD care, based on data collected over a 14-week period starting in November 2025. The results of this baseline survey, which represent the status quo of care practices in the region, will subsequently be incorporated into the planned nationwide, multicenter pilot study AmParkReg. The objectives are to identify existing care structures, regional differences, and potential deficits or oversupply. The questionnaire is based on key aspects of outpatient care in accordance with the guidelines of the German Society of Neurology and routine clinical practice. Data are collected using a standardized questionnaire implemented in the REDCap database. The survey documents routine outpatient treatment provided during regular quarterly visits in Germany. As no standardized outpatient care benchmarks have been published to date, this approach provides the first detailed description of the “real-world” outpatient PD care.

### 2.2 Population and participating practices

The target population for the survey comprised outpatient specialist neurology practices in Germany. A total of 50 interested practices were recruited in November 2025 at the annual congress of the German Society of Neurology (DGN), held from 12 to 15 November, at the QUANUP association stand. Of these, 21 practices participated in the survey, corresponding to a response rate of approximately 44%. The participating practices were intended to provide a representative picture of regional outpatient neurological care. Eligible practices were active outpatient specialist practices in neurology or neurology and psychiatry located in Germany, with at least 6 months of outpatient practice and a willingness to participate. Practices that had participated in comparable surveys within the previous 6 months or that exclusively provided private medical or non-medical services were excluded.

### 2.3 Sampling and recruitment

The randomly stratified sample comprised outpatient specialist practices in neurology and neuropsychiatry across Germany, with a majority of participating practices located in the Hamburg metropolitan region. Participants were recruited from multiple federal states. No surveys were conducted outside Germany. One questionnaire was returned by a practice located in Austria and was therefore excluded from the analysis. Practices were invited to participate via email, which included information on the objectives of the approach and data protection (Supplementary Figure S1A).

### 2.4 Questionnaire

The structured questionnaire was developed based on predefined quality indicators and a review of relevant literature. The focus was on the broad availability and simplicity of diagnostic and therapeutic measures to ensure that they could be implemented in any outpatient practice. The questionnaire comprised 20 items and used response formats such as Likert scale, yes/no questions, and open-ended items to maximize response rate while keeping the completion time under 10 min. The questionnaire used in the survey is provided in Supplementary Figures S2A–C. As the collection of invoice-related information could potentially provide sensitive insights, specific case numbers were intentionally not requested, not least to maintain a high response rate. Thematic focal points included practice structure, quality assurance, neurological examination, special motor tests, and the use of clinical rating scales, as well as open-ended diagnostic questions.

### 2.5 Data collection and management

Data were collected anonymously over a period of 14 weeks using the REDCap online platform. Alternatively, a paper version of the questionnaire could be returned by post to optimize response rates. All data were pseudonymized and stored securely.

### 2.6 Data analysis

Quantitative data were analyzed using descriptive and non-parametric methods and are presented as frequency distributions.

## 3 Results

### 3.1 Organizational structure and practice profile

**Figure 1:**
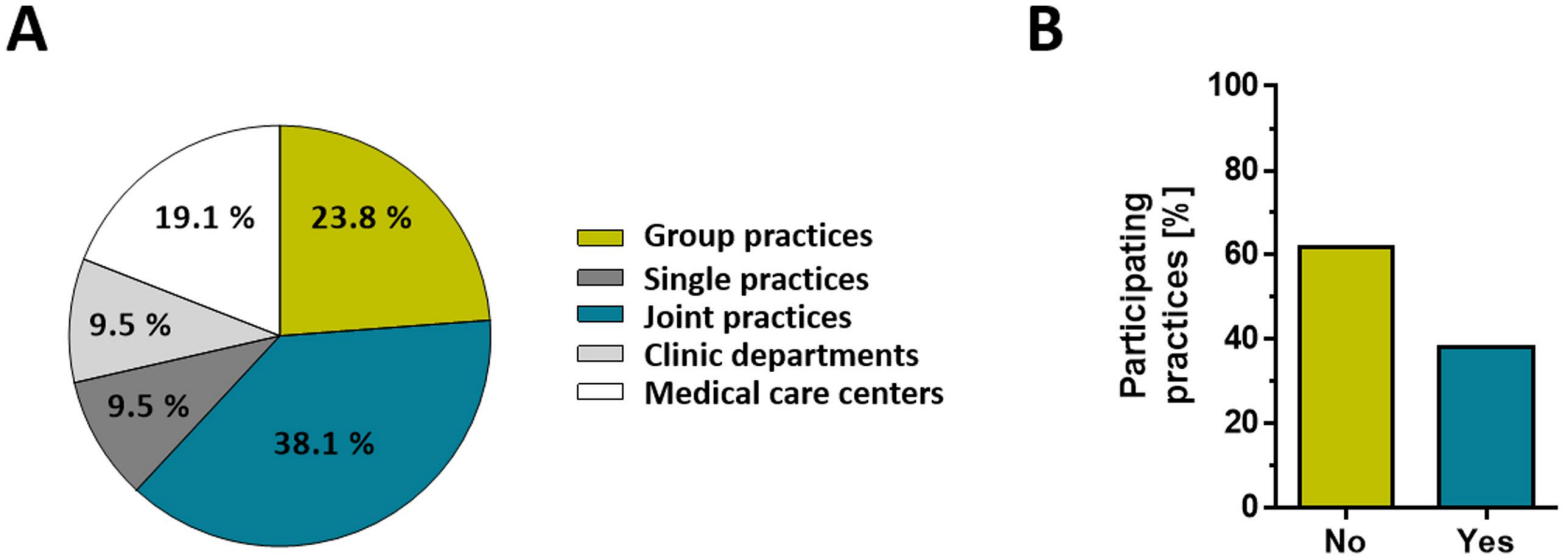
Distribution of organizational structures among practices participating in the survey **(A)** and the frequency of regularly conducted patient satisfaction surveys in these practices **(B)**. Data are presented as percentages. Panel **(B)** shows the proportion of participating practices responding “yes” or “no” to the question: “Do you conduct regular patient satisfaction surveys?”

The sample comprised 21 outpatient practices (Figure 1A) with a broadly distributed organizational profile. Most participating practices—nearly two-thirds—were organized as medical cooperatives. Specifically, 38.1% operated as joint practices, 23.8% as group practices, and 9.5% as single practices or outpatient clinic departments. In addition, 19.1% of the practices were medical care centers (Medizinische Versorgungszentren, MVZ; German term). Only a small number of practices reported a specific specialization in PD (5 of 21 practices, 23.8%). All respondents were physicians.

### 3.2 Quality management and patient feedback

**Figure 2:**
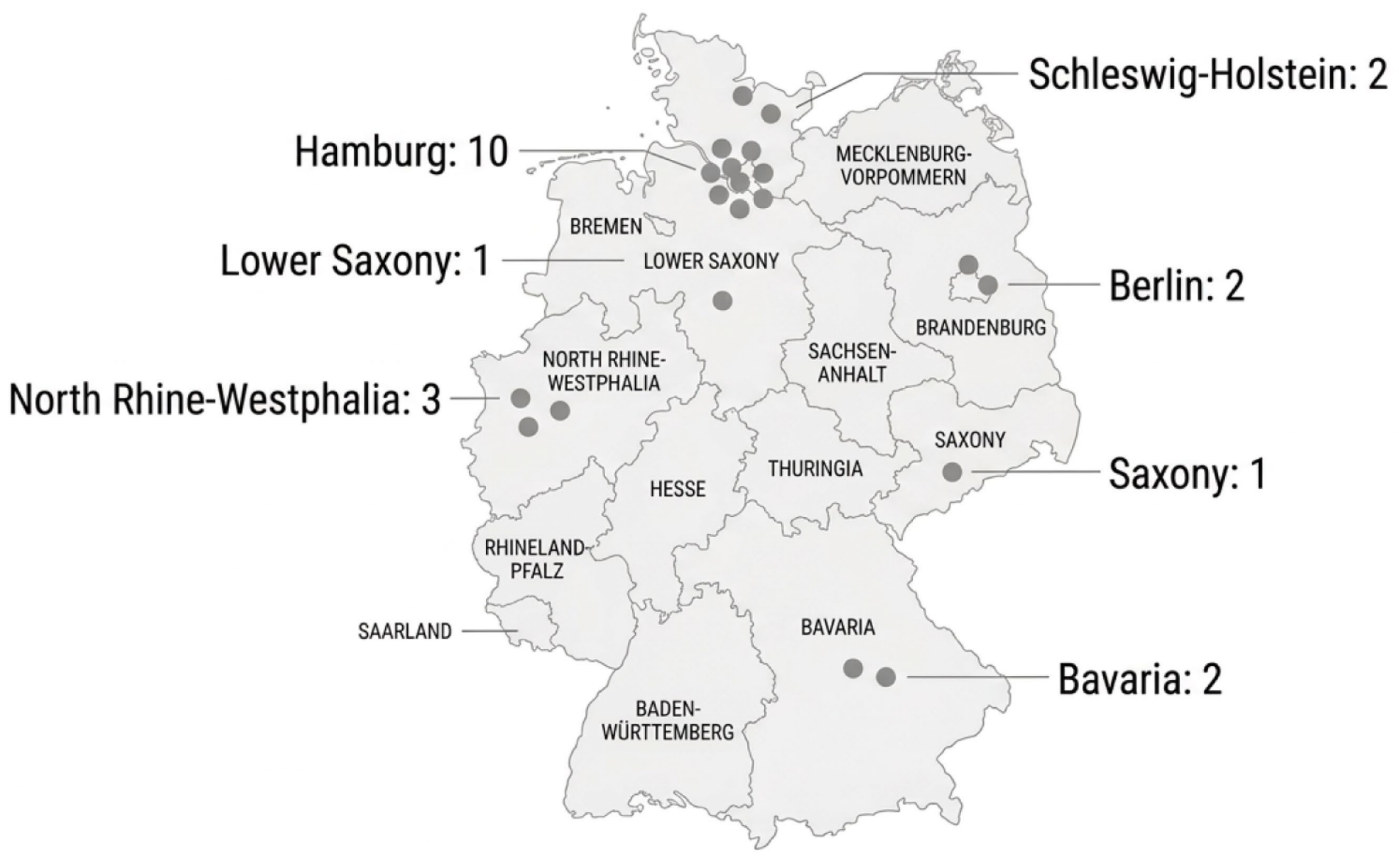
Geographic distribution of participating practices across federal states in Germany.

Among the 21 participating practices, eight (38.1%) reported routinely conducting patient satisfaction surveys (Figure 1B). In addition, respondents indicated that findings from these surveys are used to guide patient care. To further characterize the participating practices, their geographic distribution was examined (Figure 2). Practices were located in Hamburg (*n* = 10), Schleswig-Holstein (*n* = 2), Lower Saxony (*n* = 1), Berlin (*n* = 2), North Rhine-Westphalia (*n* = 3), Bavaria (*n* = 2), and Saxony (*n* = 1). Nearly half of the participating practices were located in the Hamburg metropolitan area.

### 3.3 Routine clinical observations and examinations

**Figure 3:**
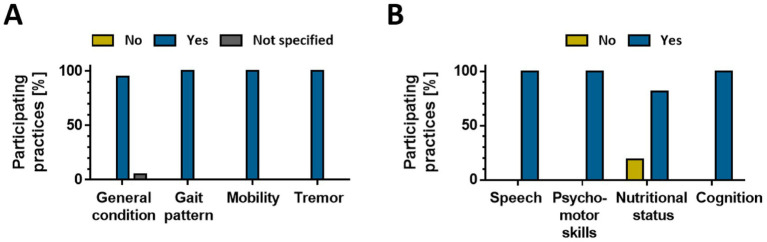
Assessments of general condition, gait pattern, global mobility, and tremor **(A)**, and non-motor symptoms **(B)** during regular outpatient visits. Data are presented as percentages of participating practices.

Visual assessment of overall condition and appearance was performed in all 21 practices. All practices reported visual assessment of gait, mobility, tremor, speech, and psychomotor features, including movement fluidity, facial expression, and gestures (Figures 3A,B). Nutritional status was assessed in 17 practices; whereas three practices did not perform such assessments. Cognitive assessment was reported by all practices, and 17 practices screened for psychosocial problems (two did not; one practice provided no information).

### 3.4 Medication, non-motor symptoms and aids

All practices routinely reviewed current medication and its effectiveness; 20 practices also routinely assessed medication-related side effects (one did not). All practices screened for non-motor functional disorders. Routine assessment of the need for medical aids was reported by 17 practices; whereas two did not perform such assessments routinely and one practice provide no information.

### 3.5 Physical examination and basic data collection

Nineteen practices reported routine physical examinations, whereas one did not. Data on height, weight and age were collected regularly by 12 practices; eight did not.

### 3.6 Standardized assessments and scales

Nineteen practices reported routinely performing rigidity examinations, whereas one did not. Reflex examinations were performed regularly in nine practices and not assessed in 11. Standardized questionnaires were routinely used by eight practices; while 12 did not use such instruments. Five practices routinely assessed non-motor symptoms using the PD non-motor symptoms (PD-NMS) questionnaire, whereas 15 did not. Similarly, five practices reported using other questionnaires, while 15 did not use any additional systematic tools. When asked to specify further examinations, respondents mentioned regular video documentation, use of the PD questionnaire-39 (PDQ-39; with 39 items), annual laboratory testing, and the timed up and go test. In addition, the wearing off questionnaire-9 (WOQ9; with 9 items), the syndrom-kurz-test (SKT), and the clock-drawing test were reported. The beck-depressions-inventory (BDI) was also mentioned.

**Figure 4:**
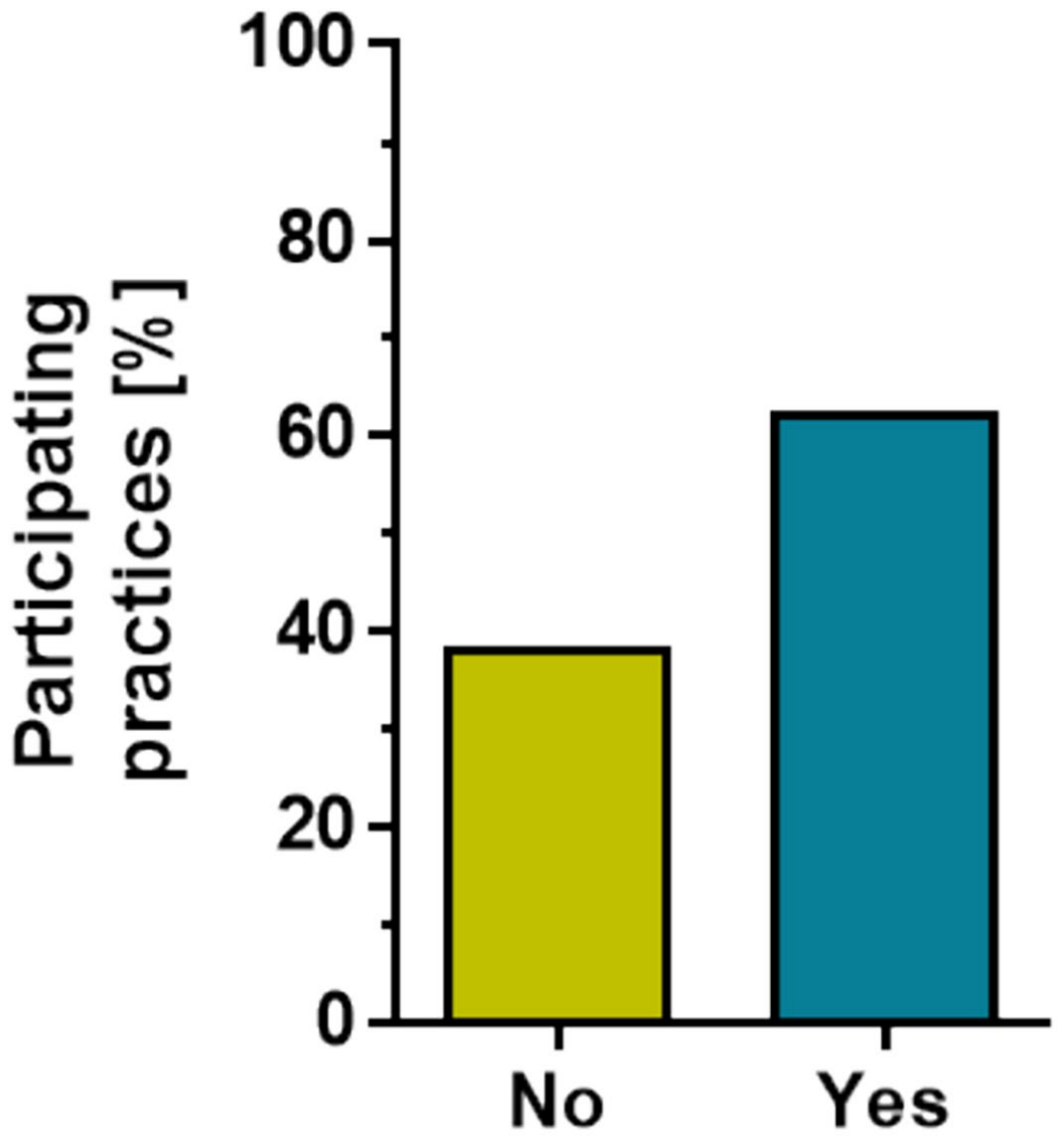
Assessment of the Hoehn and Yahr score during regular outpatient visits. Data are presented as percentage of participating practices.

The use of specific instruments varied across practices. Regular use was reported for the movement disorder society—unified PD rating scale (MDS-UPDRS), part III (motor symptoms; *n* = 7), and part IV (motor complications; *n* = 4), the montreal cognitive assessment (MoCA; *n* = 8), the PANDAS/Panda test (*n* = 5), and DemTect (*n* = 4). Five practices routinely used the PD-NMS questionnaire; whereas 15 did not. Similarly, five practices reported using other questionnaires; while 15 reported no use of additional standardized instruments. The Hoehn and Yahr scale is commonly used to assess disease stage and progression, particularly with regard to motor symptoms, postural function, gait and overall mobility across five stages. However, only approximately 60% of respondents reported regularly assessing and documenting disease stage using this scale in daily clinical practice (Figure 4).

### 3.7 Additional assessments and diagnostics

Other regularly reported assessments included video documentation, the PDQ-39, annual laboratory tests, timed up and go test, the WOQ-9, the SKT, the clock-drawing test, and the BDI. Vital signs were routinely assessed in four practices, whereas 15 practices did not assess vital signs and one practice provided no information. Six practices reported the use of additional diagnostic procedures, while 14 did not. These supplementary diagnostics included olfactory testing, handwriting or tremor spiral analysis, L-dopa- and apomorphine-challenge tests, app-based tremor analysis, autonomic testing, computed tomography and magnetic resonance imaging (CT/MRI), electrophysiological examinations, dopamine transporter (DAT) imaging, neuropsychological performance testing, sonography of the brain parenchyma and brainstem, schellong tests, and collaborative orthopedic or urological assessments. One practice noted that physical examinations were performed at baseline and thereafter as clinically indicated, rather than at every visit.

## 4 Discussion

This survey (AmParkSurvey) represents an important first step toward systematic documentation of outpatient care for people with PD in Germany and aims to provide more detailed insights into routine care in the study region. This rationale is based on the fact that outpatient PD care remains a markedly underrepresented field in the scientific literature. In contrast to the relatively extensive body of research on inpatient care, analyses of the outpatient sector have so far been scarce, have largely relied on secondary data, and generally fail to capture relevant clinical correlations (Gros et al., 2025; Dammertz et al., 2022).

### 4.1 Classification of results in context of care

Although the results presented here indicate that outpatient PD care is predominantly provided in general neurological practices without a specialization in the treatment of movement disorders, the proportion of practices specializing in PD management was relatively high in this survey, at approximately 24%. This may suggest a degree of selection bias, potentially related to the recruitment strategy (direct contact at a neurology conference) and the motivation of practices to participate in the survey. Furthermore, the fact that nearly half of the participating practices were located in city-states such as Hamburg and Berlin may partly explain the comparatively high proportion of specialized practices in the sample.

However, despite often treating substantial numbers of patients, certified PD specialist practices in Germany account for only a small proportion of overall care provision (Tönges et al., 2017; Eggers et al., 2021; Prell et al., 2020). For example, only five certified PD specialist practices are currently registered nationwide (Schwerpunktpraxen, n.d., own data from QUANUP e. V.). Previous analyses of outpatient care provided by contract physicians have demonstrated marked regional differences in the distribution of and access to care, indicating that guideline-adherent treatment is not consistently available to all patients at all times (Binder et al., 2018).

Nevertheless, it is important to emphasize (see also Supplementary Table S1 for a summary of the main results) that, despite the relatively high level of specialization among the practices participating in the survey, routine visual clinical assessment remains the central tool for clinical evaluation in everyday practice. In contrast, well-validated and standardized motor and non-motor assessment scales, such as the MDS-UPDRS, PD-NMS, or PDQ-9, as well as standardized cognitive tests (e.g., MoCA, DemTect), appear to be used primarily in scientific studies and university hospitals settings rather than in routine outpatient care. This discrepancy between guideline recommendations and everyday clinical practice, which has already been noted, is consistent with findings published in the international literature (Binder et al., 2018; Timpel et al., 2022; Eggers et al., 2021; Rosqvist and Odin, 2023; Csoti et al., 2012).

### 4.2 Strengths and weaknesses of outpatient care in relation to perspective of the survey

There is no doubt that the long-standing, continuous care provided to patients with chronic PD and their families-based on personal relationships—and the individualized treatment built on this are major strengths of outpatient care in Germany. This also represents a significant difference compared with other European countries that have different care structures (Bloem et al., 2021; Ebersbach, 2008).

On a further positive note, it should be emphasized that essential fundamental aspects of medical history and clinical assessment such as documentation of current medication, its efficacy, and side effects, as well as screening for non-motor symptoms and psychosocial stress—are firmly established in most of the practices surveyed. This suggests that the fundamental pillars of holistic care (motor skills, non- motor symptoms, psychosocial factors, need for aids) are generally addressed in daily practice, even though the degree of systematization and documentation varies. The present survey was unable to determine whether family members are regularly involved in care. However, it may be assumed that this occurs infrequently in routine practice, which is often characterized by considerable time constraints. The additional examinations reported by several practices (e.g., timed up and go, PDQ-39, olfactory testing, handwriting samples, imaging procedures, DAT-SPECT, neuropsychological tests) may indicate that more specialized or academically affiliated settings provide a broader diagnostic spectrum, including the use of standardized assessment tools. The limited availability of such specialized structures remains a recurring challenge in outpatient care.

However, it should be noted critically that only a minority of practices use these standardized scores systematically and repeatedly, and that essential elements of structured quality management—such as regular patient satisfaction surveys—are only implemented in some facilities. This heterogeneity makes it difficult to objectively assess the quality of care and to compare practices and regions. Since analyses by members of the German Parkinson’s Association (DPG) and data from the “Heathcare Atlas” (Dammertz et al., 2022) have already pointed to relevant under- and inappropriate treatments (e.g., missing or delayed medication administration, insufficient adjustment in case of complications), the observed differences in the use of structured instruments should be interpreted as a potential source of quality differences (Timpel et al., 2022; Höglinger et al., 2024; Grissinger, 2018; Saleem et al., 2023; Lance et al., 2021; Veilleux Carpentier et al., 2026).

The irregular recording of vital signs appears suboptimal, particularly given that orthostatic disorders and cardiac complications, which have a significant impact on prognosis and morbidity factors (Ziegler, 2018; Romagnolo et al., 2019), are often difficult to identify without routine standard measurements. It also remains unclear whether the regular use of standardized questionnaires to assess motor and non-motor symptoms—which, according to the present survey, is infrequent—would more effectively capture and address symptom constellations that are often decisive for quality of life (Maas et al., 2024; Martinez-Martin et al., 2011; Duncan et al., 2014). This question would require longitudinal investigation, for example within the context of the planned pilot study. Finally, it should be noted that at least some practices routinely monitor patients’ nutritional status, which is relevant given that weight loss and malnutrition increasingly become clinically significant as PD progresses (Ma et al., 2018).

### 4.3 Methodological evaluation and limitations of the study

Methodologically, the survey adopted a pragmatic, low-threshold approach to ensure sufficient willingness to participate. It was therefore necessary to design response options that were feasible and compatible with the non-academic, routine workflows of outpatient neurology practices. This approach inevitably limited the depth of the survey content. Restricting the approach to a 20-item questionnaire with predominantly closed-ended questions and a completion time of less than 10 min is reasonable from a pragmatic research perspective and likely increased participation rates (Edwards et al., 2009; Greten et al., 2025). Nevertheless, important topics such as interprofessional collaboration, digitalization, self-management, and medication management could only be addressed superficially. With a sample size of 21 outpatient practices in Germany, the findings allow at best for an initial exploratory assessment and cannot be considered representative.

This limitation is further accentuated by the fact that most participating practices were located in major cities or metropolitan regions, particularly Berlin and Hamburg, with fewer practices from less densely populated federal states and rural areas (Figure 2). Accordingly, both selection bias and self-reporting bias must be considered, including the possibility of socially desirable responses, as the reported information could not be independently verified.

Another methodological limitation is the strictly cross-sectional design. In addition, the absence of hard outcome parameters (e.g., hospitalization rates, falls, need for longterm care, mortality, or patient-reported outcome measures) precludes a direct linkage between structural and process-related variables and clinical outcomes. The present approach should therefore be regarded as a hypothesis-generating, descriptive investigation intended to serve as a foundation for further longitudinal studies, such as the planned pilot study AmParkReg.

### 4.4 Critical assessment considering existing literature

The findings of the surveys confirm known and suspected problem areas: there is a lack of mandatory quality standards (Tamás et al., 2020; Tönges et al., 2024; Howick et al., 2024), the level of specialization could be improved, and there is clear deficit in cross-sectoral and interprofessional communication structures. In international comparison—most notably with Dutch care structures—these findings point to pronounced structural and research-related deficits, which are likely to have a substantial impact on the effectiveness and quality of care (Keus et al., 2014; Maas et al., 2024; Bloem et al., 2021). As previously noted, complex PD treatment in Germany lacks clearly defined quality criteria and, consequently, corresponding reimbursement structures (Scherbaum and Tönges, 2024; Tönges et al., 2024). In the outpatient sector—with the exception of specific provisions for semi-inpatient and day-clinic care—only general guideline recommendations are available, without adequate consideration of the specific organizational and structural conditions of outpatient care (Timpel et al., 2022; Schwerpunktpraxen, n.d.). Unlike in several other European countries, easily accessible and validated standardization procedures, scoring systems, and the systematic involvement of non-medical practice staff have not yet been widely established in Germany (Keus et al., 2014; Maas et al., 2024; Bloem et al., 2021; Ebersbach, 2008).

This makes it even more essential that outpatient care structures not only manage their workload but also assess the impact of their activities and critically review the quality of outcomes (National Association of Statutory Health Insurance Physicians, 2025; Goerz et al., 2024). This is also important to demonstrate that guideline-adherent outpatient care can lead to evidence-based improvements in quality of care (Rajan et al., 2020).

### 4.5 Need for an outpatient Parkinson’s care registry

The planned registry project (AmParkReg), which builds on this initial baseline survey, could provide an opportunity to gain deeper insights into outpatient Parkinson’s care and standard management. It would thus offer the potential to define practical minimum standards and to link these with patient-relevant outcome parameters. The implementation of such a project will require a high level of motivation from all stakeholders involved—including non-specialist care providers—as well as robust scientific survey methodology, appropriate infrastructure, and adequate remuneration for participating practices, in order to avoid additional waiting times in already overburdened outpatient settings. However, evidence from international studies and published experiences suggests that even relatively simple measures, such as professional Exchange, continuing professional development, as well as standardized care and communication pathways, can substantially improve both clinical care and practice-oriented research (Keus et al., 2014; Maas et al., 2024; Bloem et al., 2021; Keus et al., 2012). The close collaboration with QUANUP e. V. and PD specialist practices may represent a methodological strength of the project, however, it also poses a potential risk if less specialized care structures and rural regions with limited infrastructure remain underrepresented.

### 4.6 Conclusion: implications for AmParkReg and future research

Despite the methodological limitations discussed here, the authors consider that this survey provides an initial and realistic impression of the strengths and weaknesses of outpatient PD care in Germany. It offers indicative insight into a high level of clinical expertise—despite structural challenges and substantial time constraints—while simultaneously highlighting structural shortcomings and areas where standardization requires improvement. The results therefore constitute a solid foundation for future longitudinal investigations, including the AmParkReg pilot study currently in preparation, which aims to contribute to improvements in the quality of care and to strengthen the evidence base for outpatient PD care in the medium to long term. Preparations for this next step are already underway, including the development of a statistical analysis plan, the establishment of the necessary IT infrastructure, and the integration of appropriate measurement tools—adapted to the outpatient setting—to enable the systematic collection and evaluation of treatment- and outcome-relevant parameters. In addition, it would be desirable to enable meaningful comparisons of national outpatient data with international results and to facilitate the exchange of transferable findings.

## Data Availability

The original contributions presented in the study are included in the article/Supplementary material, further inquiries can be directed to the corresponding author.

## Glossary

AmParkReg: Ambulatory Parkinson Registry
AmParkSurvey: Ambulatory Parkinson Survey
BDI: Beck Depression Inventory
CT: Computed Tomography
DAT: Dopamine Transporter
DAT-SPECT: Dopamine Transporter Single-Photon Emission Computed Tomography
DemTect: Dementia Detection Test
DPG: Deutsche Gesellschaft für Parkinson und Bewegungsstörungen e.V. (German Parkinson’s Association)
eCRF: Electronic Case Report Form
ePROMs: Electronic Patient-Reported Outcome Measures
e.V.: (Eingetragener Verein)-Registered association
GCP: Good Clinical Practice
IT: Information Technology
L-dopa: Levodopa
MDS-UPDRS-III: Movement Disorder Society – Unified Parkinson’s Disease Rating Scale, Part III
MDS-UPDRS-IV: Movement Disorder Society – Unified Parkinson’s Disease Rating Scale, Part IV
MoCA: Montreal Cognitive Assessment
MRI: Magnetic Resonance Imaging
MVZ: (Medizinisches Versorgungszentrum)-Medical Care Center
n: Number
NMS: Non-Motor Symptoms
NMSQuest: Non-Motor Symptoms Questionnaire
NMSScale: Non-Motor Symptoms Scale
OWL: (Ostwestfalen-Lippe)-East Westphalia-Lippe
PANDA/Panda test: Parkinson Neuropsychometric Dementia Assessment
PD: Parkinson’s Disease
PD-MSS: Parkinson’s Disease – Motor or Non-Motor Symptom Scale
PD NMS Questionnaire: Parkinson’s Disease Non-Motor Symptoms Questionnaire
PDQ-39: Parkinson’s Disease Questionnaire-39
PKB/ PKT: (Parkinson-Komplexbehandlung/Parkinson-Komplextherapie)-Parkinson Complex Therapy
QM: (Qualitätsmanagement)-Quality Management
QUANUP e. V: (Verein für Qualitätsentwicklung in Neurologie und Psychiatrie)-Association for Quality Development in Neurology and Psychiatry
REDCap: Research Electronic Data Capture
SKT: Syndrom-Kurztest
WOQ-9: Wearing-Off Questionnaire-9
