# Supplementary material for: Searching for a needle in a haystack—ambulatory research in Parkinson’s disease in Germany
Source: Front Aging Neurosci. 2026 Jun 10;18:1820262. doi: 10.3389/fnagi.2026.1820262 (PMC13290942; doi:10.3389/fnagi.2026.1820262)
Supplement: Supplementary file 1 [file Data_Sheet_1.pdf]

Supplementary Material

Searching for a needle in a haystack – Ambulatory research in Parkinson`s disease in Germany

Ingmar Wellach [1, 2, 3, 4], **Dirk Becker** [1, 3, 4], **Kristina Schmidt** [1, 3], **Isabel Doblinger** [1, 3], **Susann Eichler** [5], **Lisa Hillmer** [5], **Honorine Atanga** [2, 5] and **Christoph Redecker** [1, 2, 6]

[1]Association for Quality Development in Neurology and Psychiatry (QUANUP e.V.), Hamburg, Germany. [2]Bielefeld University, Medical School and University Medical Center OWL, Hospital Lippe, Department of Neurology and Neurogeriatrics, Lemgo, Germany. [3]Neurology and Psychiatry Practice Hamburg Walddoerfer, Hamburg, Germany. [4]Evangelical Amalie Sieveking Hospital, Hamburg, Germany. [5]Bielefeld University, Medical School and University Medical Center OWL, Hospital Lippe, Department of Science and Academic Medicine, Detmold, Germany. [6]Neuropraxis Detmold, Detmold, Germany.

Supplementary Figure S1A. Recruitment of practices via email invitation, providing information on the objectives of the approach and data protection.

Confidential

Page 1

Survey on Treatment Practices for Patients with Parkinson's Disease

Survey among community neurologists on the topic:

Treatment routines for patients diagnosed with Parkinson's.

Dear Colleague,
Dear Colleague,

We cordially invite you to participate in our survey regarding treatment routines for patients with Parkinson's disease.

Purpose of the Survey:

Your responses will help us plan an extensive cohort study on Parkinson's treatment, which we aim to conduct with around 30 independent medical practices.

We kindly ask you to participate in this survey to help us prepare for our study. Completing the questionnaire should take no more than 5 to 10 minutes of your time.

If you are interested in participating in this cohort study, please contact Ms. Doblinger and Ms. Schmidt via email at studien@quanup.de

Your participation in this survey is completely voluntary. You may choose not to participate or stop at any time without providing a reason. No personal information is required, and the survey is anonymous and will only be conducted once.

By filling out the questionnaire, you agree to take part in the survey and confirm that you have been informed about its purpose. You also consent to the further processing of your responses by staff members at Quanup and the University Hospital OWL.

Your data will be kept confidential and will not be shared with third parties. The questionnaire will be stored for up to 10 years and then destroyed. Please note that the results of the survey may be published in medical journals, but your identity will remain anonymous.

Your Right to Ask Questions:
If you have any questions about this survey or the planned study, please feel free to contact us.

Quanup e.V. c/o Neurology & Psychiatry Practice
Hamburg Walddörfer
Wiesenkamp 22c
22359 Hamburg
Phone: 0176/56 76 004
Email: studien@quanup.de

I have read the information above and consent to the use of my data.	○ Yes
I am authorized by the practice owner to provide this information.	○ Yes

You will be forwarded to the survey once you have answered "yes" to both questions.

2025-10-01 09:26 projectredcap.org REDCap

Supplementary Figure S2A. Questionnaire used in the survey.

Confidential

Parkinson's Treatment Routine Questionnaire

1 Practice Name – NOTE: Voluntary information ______________________

2 Type of Practice
- ○ Solo Practice
- ○ Medical Center (MVZ)
- ○ Group Practice
- ○ Practice Community
- ○ Hospital
- Other: ______

3 Specialty (Multiple selections possible)
- ☐ Neurology
- ☐ Neuropsychiatry
- ☐ Psychiatry
- ☐ Other
- ☐

4 Specialization ______________________

5 In which federal state is your practice located? ______________________

6 Who is completing this questionnaire?
- ○ MFA
- ○ Physician
- ○ Study Nurse
- ○ Other

The following examinations are routinely performed in my/our practice:

7 Do you regularly conduct patient satisfaction surveys?
- ○ Yes
- ○ No
- ○ No response

7a Do the survey results have an impact on how patients are cared for?
- ○ Yes
- ○ No
- ○ No response

8 Visual assessment of overall condition – evaluating the patient's general appearance
- ○ Yes, performed routinely
- ○ No, usually not performed
- ○ No response

9 Visual assessment of walking pattern
- ○ Yes, performed routinely
- ○ No, usually not performed
- ○ No response

10 Visual assessment of mobility
- ○ Yes, performed routinely
- ○ No, usually not performed
- ○ No response

11 Visual assessment of tremor
- ○ Yes, performed routinely
- ○ No, usually not performed
- ○ No response

12 Assessment of speech
- ○ Yes, performed routinely
- ○ No, usually not performed
- ○ No response

Supplementary Figure S2B. Questionnaire used in the survey.

Confidential

13	Assessment of psychomotor function (fluidity of movements/facial expressions/gestures, etc.)	○ Yes, routinely performed<br>○ No, generally not performed<br>○ No information provided
14	Assessment of nutritional status	○ Yes, routinely performed<br>○ No, generally not performed<br>○ No information provided
15	Cognitive assessment	○ Yes, routinely performed<br>○ No, generally not performed<br>○ No information provided
16	Conducting an interview: Screening for psychosocial issues:	○ Yes, routinely performed<br>○ No, generally not performed<br>○ No information provided
17	Recording medications and their effectiveness	○ Yes, routinely performed<br>○ No, generally not performed<br>○ No information provided
18	Checking for side effects	○ Yes, routinely performed<br>○ No, generally not performed<br>○ No information provided
19	Screening for non-motor symptoms	○ Yes, routinely performed<br>○ No, generally not performed<br>○ No information provided
20	Assessing the need for therapeutic and assistive devices	○ Yes, routinely performed<br>○ No, generally not performed<br>○ No information provided
21	Physical Examination:	○ Yes, routinely performed<br>○ No, generally not performed<br>○ No information provided
22	Assessment using the Hoehn & Yahr Scale	○ Yes, routinely performed<br>○ No, generally not performed<br>○ No information provided
23	Rigidity Examination	○ Yes, performed routinely<br>○ No, generally not performed<br>○ No information provided
24	Reflex Assessment	○ Yes, routinely performed<br>○ No, generally not performed<br>○ No information provided
25	Are questionnaires used?	○ Yes, routinely performed<br>○ No, generally not performed<br>○ No information provided

Supplementary Figure S2C. Questionnaire used in the survey.

2 If so, which questionnaires?	☐ UPDRS I-IV<br>☐<br>☐ MoCA<br>☐ PANDA<br>☐ DemTec<br>☐ PD NMS<br>☐ Other: ______
26 Recording of vital signs (blood pressure, pulse)	○ Yes, routinely performed<br>○ No, generally not performed<br>○ No information
27 Would you like to provide details about other diagnostics or add any comments?	○ Yes<br>○ No
28 Please share any other diagnostic information or additional remarks	______________________

Supplementary Material

Supplementary Table S1. Summary of the main results**.**

**A** Section	**B** Key findings
**Methods**	• Cross-sectional study to conduct the first-ever survey of undefined standards in outpatient Parkinson's care<br>• Basis for the nationwide multicenter pilot study AmParkReg<br>• Focus: mapping regional care structures, differences, deficits or over-provision<br>• Data collection via standardised REDCap questionnaire, anonymous<br>• Data analysis: descriptive, non-parametric (frequency distributions) practising specialists in neurology or neurology & psychiatry in Germany<br>• Recruitment: DGN Congress (12–15 Nov. 2025), QUANUP stand<br>• Inclusion criteria: at least 6 months' outpatient practice,<br>• willingness to participate<br>• Exclusion: private medical or non-medical services, participation in similar surveys in the last 6 months
**Target group & sample**	• Sample: 21 practices (response rate ~44%)
**Practice structure**	• ~38% joint practices, ~24% group practices, ~10% solo practices or hospital outpatient clinics, ~20% medical care centres<br>• 4 practices specialising in Parkinson's disease<br>• All respondents were physicians
**Geographical distribution**	See **Figure** 2
**Patient assessments**	• 8 practices routinely conduct patient satisfaction surveys<br>• Results of these surveys are incorporated into patient care
**Clinical observation & examination**	• 21 practices: Routine assessment of gait, tremor, speech, psychomotor function, facial expressions, gestures
**Nutritional status**	• Nutritional status: 17 out of 21 practices assess this regularly
**Cognitive tests**	• Cognitive tests in all practices
**Psychosocial problems**	• Psychosocial problems: 17 practices assess these regularly
**Medication & non-motor symptoms**	• All practices regularly review medication and efficacy<br>• 20 practices ask about side effects; all record non-motor symptoms<br>• 17 practices regularly assess the need for medical aids
**Physical examination**	• 19 practices: regular physical examination<br>• Routine rigidity assessment: 19 practices<br>• Reflex testing: 9 regularly, 11 did not<br>• Vital signs: only 4 practices check these regularly<br>• In some cases, baseline examination and follow-up examinations as clinically indicated

Supplementary Material

Supplementary Table S1. Summary of the main results (continued)

**A** Section	**B** Key findings
**Baseline data**	• Height, weight, age: 12 practices document these regularly
**Standardised questionnaires & scales**	• 8 practices use standardised questionnaires<br>• 5 practices use the PD-NMS questionnaire (non-motor symptoms), 15 do not<br>• 5 others use additional questionnaires or tests: video analysis, PDQ-39, Timed Up and Go, WOQ-9, SKT, clock test, BDI<br>• MDS-UPDRS-III (n=7), MDS-UPDRS-IV (n=4), MoCA (n=8), PANDAS/Panda (n=5), DemTect (n=4)<br>• Hoehn & Yahr scale: ~60% document regularly
**Additional diagnostics & special tests**	• 6 practices use additional diagnostic procedures<br>• These include: smell test, handwriting/spiral analysis, L-dopa/apomorphine test, app-based tremor analysis, autonomic tests, CT/MRI, electrophysiology, DAT scan, neuropsychological tests, brain parenchymal sonography,<br>• Schellong test, orthopaedic/urological co-assessment
